# Surgical anesthesia with a combination of T12 paravertebral block and lumbar plexus, sacral plexus block for hip replacement in ankylosing spondylitis: CARE-compliant 4 case reports

**DOI:** 10.1186/s12871-017-0358-7

**Published:** 2017-06-26

**Authors:** Xijian Ke, Ji Li, Yong Liu, Xi Wu, Wei Mei

**Affiliations:** Department of anesthesiology and Pain medicine, Tongji Hospital, Tongji Medical College, Huazhong University of Science and Technology, 1095 Jiefang Road, Wuhan, 430030 People’s Republic of China

**Keywords:** Case report, Paravertebral block, Lumbar plexus block, Sacral plexus block, Ankylosing spondylitis, Total hip arthroplasty

## Abstract

**Background:**

Anesthesia management for patients with severe ankylosing spondylitis scheduled for total hip arthroplasty is challenging due to a potential difficult airway and difficult neuraxial block. We report 4 cases with ankylosing spondylitis successfully managed with a combination of lumbar plexus, sacral plexus and T12 paravertebral block.

**Case presentation:**

Four patients were scheduled for total hip arthroplasty. All of them were diagnosed as severe ankylosing spondylitis with rigidity and immobilization of cervical and lumbar spine and hip joints. A combination of T12 paravertebral block, lumbar plexus and sacral plexus block was successfully used for the surgery without any additional intravenous anesthetic or local anesthetics infiltration to the incision, and none of the patients complained of discomfort during the operations.

**Conclusions:**

The combination of T12 paravertebral block, lumbar plexus and sacral plexus block, which may block all nerves innervating the articular capsule, surrounding muscles and the skin involved in total hip arthroplasty, might be a promising alternative for total hip arthroplasty in ankylosing spondylitis.

## Background

Ankylosing spondylitis (AS) is a chronic and progressive autoimmune disease [1]. It mainly affects the sacroiliac joints and axial skeleton and at late stage causes fusion and rigidity of the spine and massive joints [2]. Many patients present difficult airway due to immobilization of the neck and temporomandibular joints which makes intubation general anesthesia less preferable. Because of ossification of the ligament and deformity of the lumbar spine, success rate of epidural and spinal anesthesia reported in patients with ankylosing spondylitis was very low [3]. Several studies have reported combination of lumbar plexus and parasacral plexus for hip surgeries [4–8]. In these studies, however, either large dose of propofol, iliac crest block [9] or infiltration to the incision [4] was needed. We report here with a novel combination of lumbar plexus block, sacral plexus block and T12 paravertebral block successfully used for total hip arthroplasty for 4 patients with severe ankylosing spondylitis without any opioids and intravenous propofol. Ethical approval of this report (TJ-C20160106) was given by the medical ethics committee of Tongji Hospital of Huazhong University of Science and Technology.

## Case presentation

**Fig. 1:**
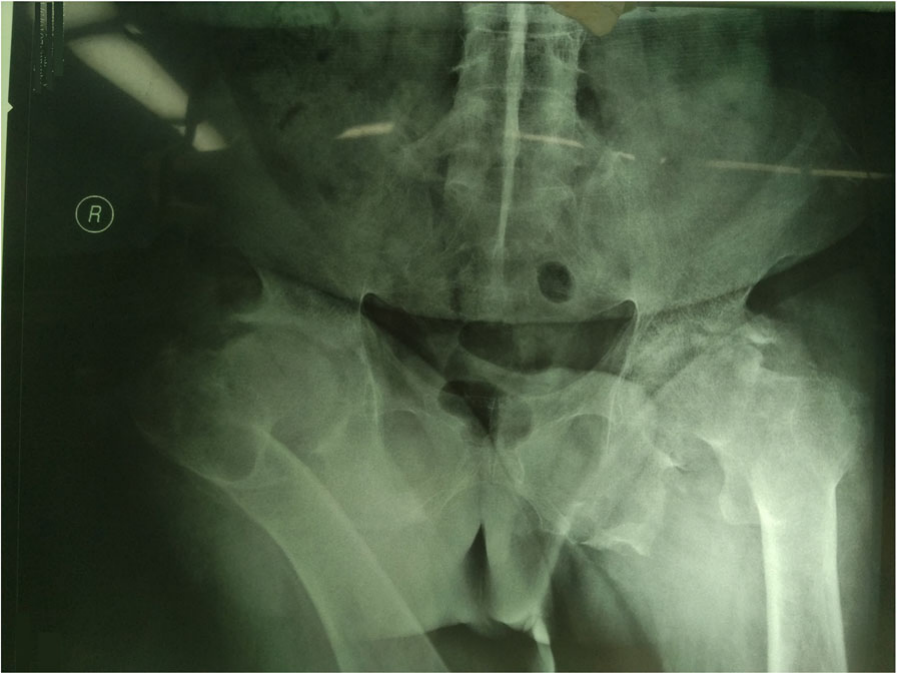
Radiograph of lumbo-sacral vertebrae and pelvis shows bamboo like bony bridge, narrowed intervertebral space, fusion of both sacroiliac joints and extreme deformity of both hip joints

**Case 1:** A 38-year-old male (weight 75 kg, ASA status III) was scheduled for right total hip arthroplasty (THA) due to severe bilateral rigid and sore hip joints. He had a history of ankylosing spondylitis for more than 18 years. Despite a long term usage of sulfasalazine and “painkiller”, the patient developed a rigid kyphotic and scoliotic deformity. An overall physical examination revealed rheumatoid arthritis involved most articles in the body resulting in limited movement and swelling of interphalangeal joints, metacarpophalangeal joints, elbow joints, shoulder joints, and ankle joints, with rigid bilateral hip and knee joints preventing any walk. The airway assessment revealed a Mallampati class III, interincisor distance of 3 cm, and rigid neck incapable of any movement. X-ray of his cervical, thoracic and lumbar vertebrae and pelvis revealed a loss of physiological curvature of the vertebral column, kyphosis, hyperplasia at the edge of the vertebrae, “bamboo” like bony bridge, blurred facet joints, and calcification of ligament, a fusion of both sacroiliac joints, rough articular face and narrowed articular cavity and hyperplasia of both hip joints (Fig. 1). After administration, he received immunosuppressive therapy, with prednisone 20 mg per day, and methotrexate 10 mg once per week, and anti-TNF therapy with etanercept (TNF receptor fusion protein) 25 mg i.h. twice per week, and anticoagulation therapy with low-molecular-weight heparin 4000 U per day.

**Case 2:** A 61 years old male (weight 75 kg, ASA status III) suffered from AS for 20 years, and was scheduled for left total hip arthroplasty (THA). He had a history of coronary heart disease and received coronary stent implantation 7 years ago. A physical examination revealed rigidity of cervical and lumbar spine, severe press pain and immobilization of left hip joints. An X-ray of the neck, chest and lower back revealed bamboo-like changes of the spine. CT of the hip joints reported necrosis of the left femoral head, and ankylosis of left hip joint. He had taken aspirin for anticoagulation therapy for a long term, and was replaced by low molecular weight heparin.

**Case 3:** A 26 years old female (weight 47 kg, ASA status II) was scheduled for left total hip arthroplasty. She was diagnosed with ankylosing spondylitis 14 years ago, with pain on almost all major joints of her body. She could not stand or walk because of bilateral femoral head necrosis. She had a family history of ankylosing spondylitis, with her father also suffered from this disease. Physical examination revealed rigidity of neck and left hip joint. X-ray of the thoracic and lumbar vertebrae reported rigidity of the spine, calcification of interspinous ligament. X-ray of bilateral sacra-iliac joints, hip joints, knees and ankles reported narrow joint space, osteoproliferation, bone defect and joint deformity. The chest X-ray reported texture enhancement of bilateral lungs and infection of right lower lobe.

**Case 4:** A 65 years old male (weight 76 kg, ASA status III) was scheduled for left THA. He was diagnosed with AS for 10 years, and developed necrosis of both femoral heads, and could not walk because of pain in the left hip joint. He had a history of hypertension for 10 years, and diabetes mellitus for 7 years, and smoking history for 50 years with nearly 50 cigarettes per day. A chest X-ray revealed enhanced texture of bilateral lungs and right pleural effusion. X-ray of his pelvic revealed bilateral femoral head necrosis, narrowed joint space, osteoproliferation and bone defect under articular surface of bilateral sacra-iliac joints and hip joints, and bamboo-like changes of lower lumbar spine.

### Ultrasound guided T12 paravertebral block, lumbar plexus block and sacral plexus block

In the operation room, the patients were settled in a lateral decubitus position with surgical side upmost, and were given oxygen through a face mask. 500 ml of Ringer's solution was infused while pulse oximeter, NIBP and ECG monitors were connected. Difficult intubation carts, including LMA, bougie, fibre optic bronchoscopy, cricothyrotomy needle and tracheostomy set, were standby.

**Fig. 2:**
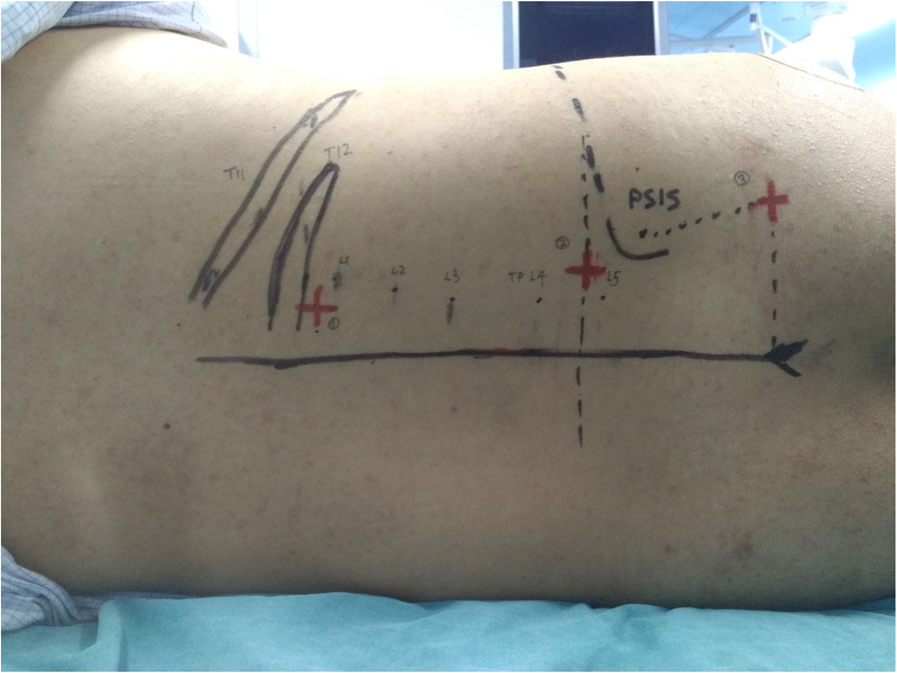
The lateral decubitus position and surface anatomic landmarks

We performed all of the peripheral nerve blocks guided by the ultrasound (M-Turbo, Sonosite, USA) combined with nerve stimulator (Stimuplex HNS12, B-Brawn, USA), with the patient in the same lateral decubitus position (Fig. 2), and we chose a 10 cm 22G needle (Sonoplex, Pajunk, Germany).

**Fig. 3:**
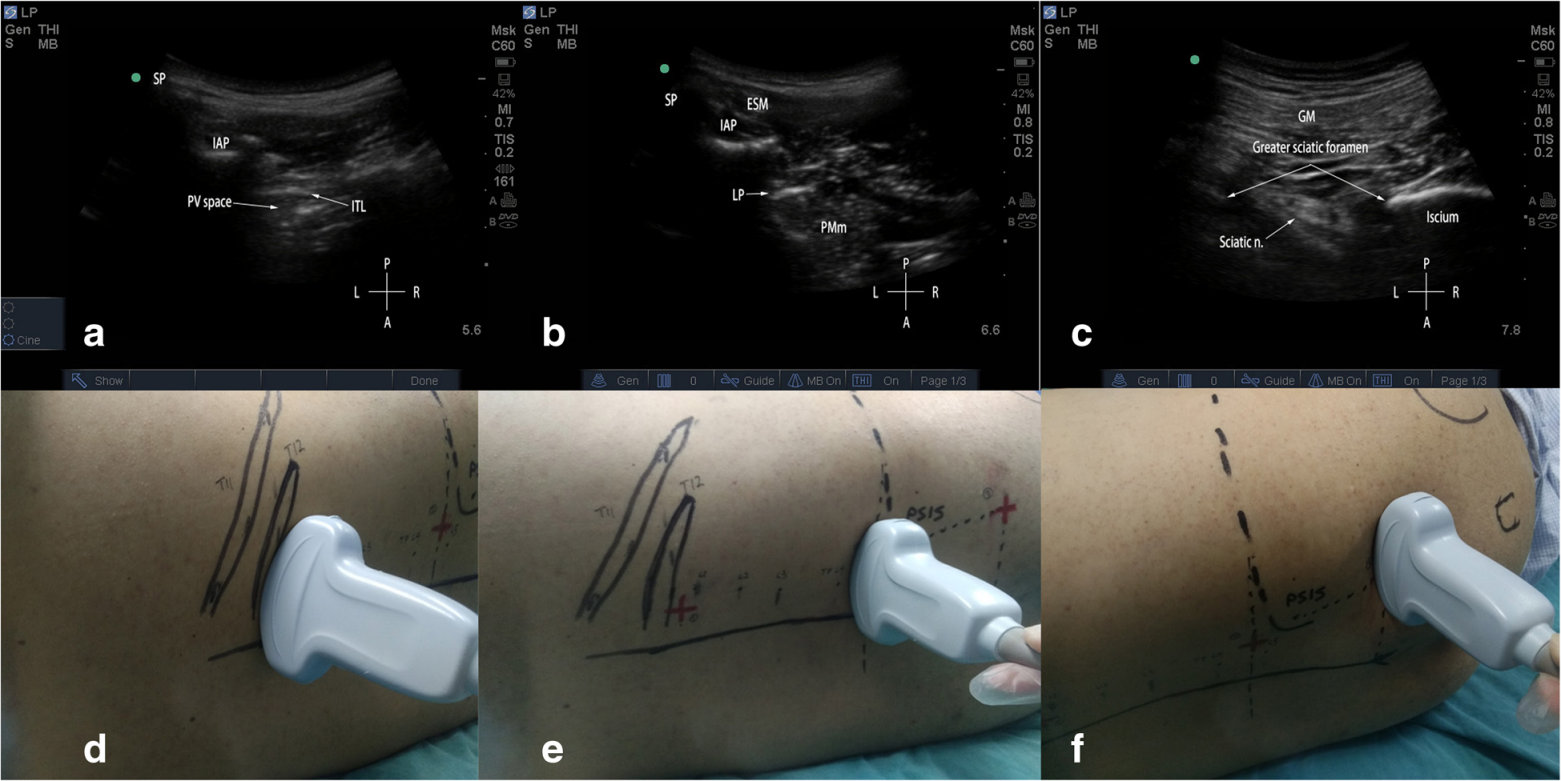
Ultrasonography and the corresponding positions of transducer. **a**: ultrasonography of T12 paravertebral space, when ultrasound probe was positioned as in panel **d**; **b**: ultrasonography of lumbar plexus when the ultrasound probe was positioned transversely as in panel **e**; **c**: ultrasonography of sacral plexus when ultrasound probe was positioned transversely at the level of sacral hiatus as in panel **f**. AP = articular process; SP = spinous process; ITL = intertransverse ligament; PV space = paravertebral space; ESM = erector spinae muscle; LP = lumbar plexus; PMm = psoas major muscle; GM = gluteus maximus muscle; sciatic n. = sciatic nerve; P = posterior; A = anterior; L = left, R = right

We performed T12 paravertebral block via a transversal in-plane technique at the level of inferior articular process, as previously described by Krediet, A.C., et al. [10] A low-frequency ultrasound transducer was placed parallel to the midline for a sagital scan to identify the 12^th^ rib, and then rotated at the level that showed the 12^th^ rib connecting to its corresponding transverse process and creating an acoustic shadow (Fig. 3d). Scanning slightly inferior, to show the “thumb-like” transverse process, the thoracic paravertebral space is located anterior to the transverse process but is obscured from vision. More inferior to the transverse process, the TPV space can be visualized with ultrasound and is no longer shielded by bone (Fig. 3a). The contour of inferior articular process and acoustic shadow constitute the medial boundary of the TPV space. The intertransverse ligament and the diaphragm form the posterior and anterior boundary respectively. After skin antisepsis, sterile draping was placed and ultrasound probe was sheathed. The needle was inserted lateral to the probe and advanced in-plane from lateral to medial, to penetrate the intertransverse ligament, then slightly redirected and advanced further to a final position lateral to the vertebral body where a stimulating current with 0.5–0.8 mA induced appropriate abdominal muscle twitch. And 10 ml 0.4% ropivacaine was injected in incremental doses with negative aspiration throughout.

For lumbar plexus block, the low-frequency ultrasound probe was placed along the Tuffier’s line, lateral to spinous process, to achieve an axial scan of the psoas major muscle (Fig. 3e). At the level displaying the transverse process, the probe was slid slightly cranially to show a hyperechoic structure buried in the back portion of the psoas major muscle under transverse process, which was exactly the lumbar plexus nerves (Fig. 3b). A skin wheel was made and the needle was inserted to the right side of the probe, targeting the lumbar plexus nerves beneath transverse process, using an “in-plane” technique. We used the nerve stimulator to confirm the needle's correct position by a quadratus femoris twitch at a current within 0.5–0.8 mA, followed by 30 ml 0.4% ropivacaine that was slowly injected in 5 ml increments.

**Fig. 4:**
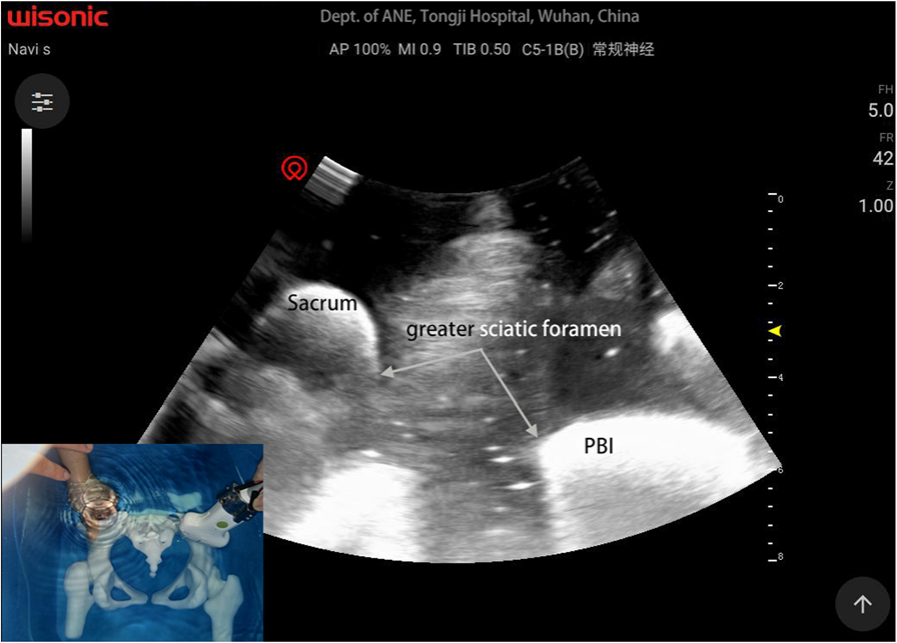
Ultrasonography of a 3D-printed pelvic phantom bathing in water, to mimic the greater sciatic foramen. PBI = posterior border of ischium

**Fig. 5:**
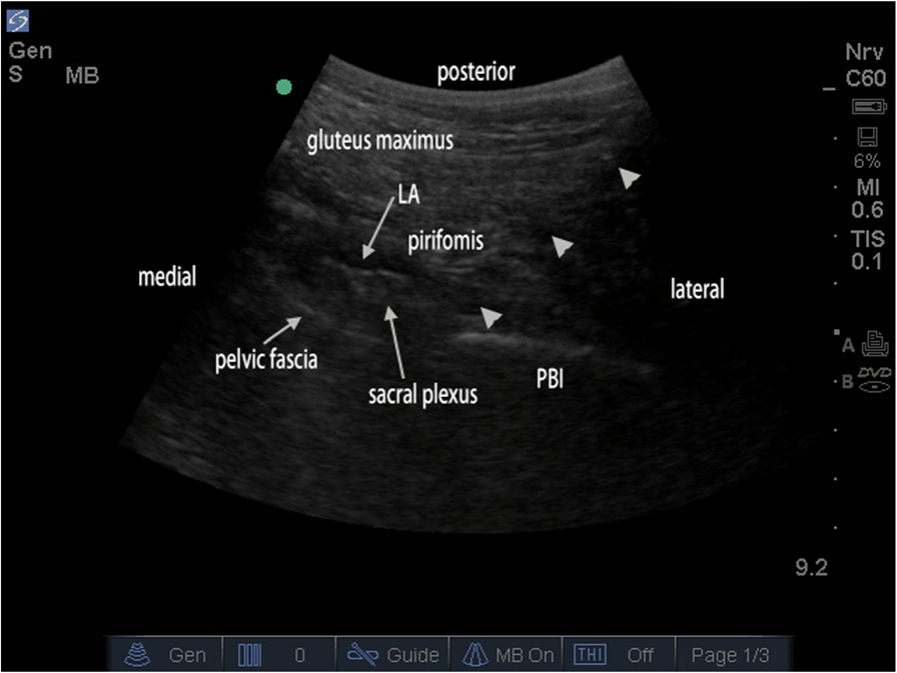
Ultrasonography of the sacral plexus bathing in local anesthetic deep to the pirifomis muscle and medial to the PBI. (LA = Local anesthetic, PBI = posterior border of ischium, triangle indicates the needle)

For sacral plexus block, we used a modified technique described by Taha [11]. The low-frequency ultrasound probe was placed transversely at the level of sacral hiatus lateral to the midline (Fig. 3f), where the posterior border of ischium (PBI) appears as a clear hyperechoic line. Alternatively, the probe was moved cranially to find the ala of the ilium that demonstrated as a continuous hyperechoic line, and then slowly slid the probe caudally until a gap that representing the greater sciatic foramen started to appear. At this point, the PBI and greater sciatic foramen could be clearly identified, which also can be clearly depicted in a 3D-print pelvic phantom (Fig. 4). The sacral plexus appeared as a hyperechoic structure located medial to the PBI (Fig. 3c) and deep to the piriformis. A skin wheel was placed and the needle was inserted lateral to the probe via an “in-plane” approach targeting the target nerve, other than “out-of-plane’ approach recommended by Taha [11]. When hamstring, leg, or foot twitches were elicited within a current of 0.5 to 0.8 mA, 20 ml of 0.4% ropivacaine was slowly injected in 5 ml increments to surround the target nerve under ultrasound monitoring (Fig. 5).

Twenty minutes later, sensory loss to pinprick had been achieved between T10 and S3 unilaterally (Table 1), while the assessment of motor function was not possible because of rigid joints. About 30 min later, the surgeries began, using a lateral-posterior approach with the same lateral decubitus position. The upmost of the incision lay about 5 cm craniomedial to the greater trochanter. The patients were given 1 mg midazolam for sedation and did not require any opioids and propofol and incision infiltration. They did not complain of pain during surgery and remained quiet but arousable throughout. For the third case, blood pressure tended to decrease during the surgery, and intravenous phenylephrine pump were required. The surgeries lasted for 38–57min with blood loss of 200–250 ml (Table 2).

**Table 1:** Assessment of sensory loss to pinprick achieved

	20 min after blocks	At the end of operation	Before leaving PACU
Case 1	T11-S2	T11-S2	T12-S2
Case2	T12-S2	T12-S2	T12-S1
Case 3	T10-S3	T11-S3	T11-S2
Case 4	T11-S2	T12-S2	T12-S2

**Table 2:** Intraoperative parameters

	Blood pressure(mmHg)	Heart rate(bpm)	SpO_2_(%)	Duration of operation(min)	Blood loss(ml)
Case 1	135–160/64–89	90–102	92–100	50	200
Case2	120–150/70–91	75–90	96–100	57	200
Case 3	83–90/46–54	65–77	95–100	38	150
Case 4	92–120/50–70	65–75	97–100	53	250

In the PACU, all the patients did not complain of discomfort in the surgical side, and the visual analog scale scores were 0–1. The patients were given a patient-controlled intravenous analgesia for 24 h postoperatively, and were discharged 7–10 days later uneventfully.

## Discussion

Ankylosing spondylitis is a common inflammatory rheumatic disease that affects the axial skeleton, causing characteristic inflammatory back pain, which can lead to structural and functional impairments and a decrease in quality of life [2]. The disease predominantly affects young men, beginning most often in the third decade [3]. Structural changes are mainly caused by osteoproliferation. Spinal stiffness, loss of spinal mobility, syndesmophytes and ankylosis are the characteristic features of ankylosing spondylitis. The hip and shoulder joints become affected in about 20% of patients with this disease. Hip involvement and extra-articular manifestations indicate poor prognosis [12]. Limited mouth opening was due to temporomandibular joint involvement was reported in 10% of patients and may increase in the late stage [1]. Involvement of the costovertebral joints may result in respiratory complications include upper lobe fibrosis and reduced chest expansion [1]. The objectives of treatment are to improve the quality of life as well as to prevent subsequent clinical deterioration [13]. Joint replacement has to be considered in patients with radiographic evidence of advanced hip involvement that have refractory pain and disability [14].

Anesthesia management for ankylosing spondylitis is a challenge due to management of difficult airway, respiratory and cardiovascular complications, as well as the medications for disease and pain control [1]. Both airway management and neuraxial access may prove to be difficult [3].

At present, general anesthesia is administered for most of the patients with ankylosing spondylitis undergoing hip surgeries. Fixed neck may make regular endotracheal intubation and tracheostomy difficult [1]. In patients with chronic cervical kyphosis, risk of neurological injury increased with excessive neck extension. Due to an anticipated difficult airway, awake fibrotic endotracheal intubation employed may subject the patients to significant discomfort. Because the fixed angle of the oropharynx axis, it may be difficult to place and secure a laryngeal mask in suitable place in patients with severe flexion deformities [1]. Furthermore, a recent large population-based study, that included 104, 088 geriatric patients undergoing hip repair surgeries, concluded that compared with neuraxial anesthesia, general anesthesia were associated with higher incidences of and risks for the adverse in-hospital outcomes of stroke, respiratory failure, and death [15].

An update to evidence-based guidelines for the management of hip fractures in older persons suggests that regional anesthesia is recommended for most patients with a grade A of evidence [16]. However spinal and epidural anesthesia are technically difficult and the patients may be subjected to an increased risk of complications. Placement of spinal needle is difficult in patients with AS due to ossification of interspinous ligaments and ligamentum flavum, and bony bridges, especially through a midline approach [17]. A 10 years review in Vancouver Hospital showed of 82 procedures performed on AS patients, 3 spinal anesthesia failed in 13 (23.8%) and epidural anesthesia was unsuccessful in every attempt [3]. Wulf reported in a comprehensive review five out of 51 patients with spinal hematoma occurred in patients with AS [18]. These may result from difficult or traumatic insertions, concurrent NSAIDs therapy and a narrow epidural space.

Disadvantages of spinal anesthesia may include urinary retention, hypotension, and issues involving perioperative anticoagulation [8], the risk of spinal hematoma, meningitis, or spinal abscess. A prospective randomized study demonstrated that hypotension induced by plain bupivacaine spinal anesthesia was found to be longer lasting and of large magnitude compared with combined lumbar/sacral plexus block [5]. Urinary tract catheterization followed by spinal anesthesia may increase the incidence rate of urinary tract infection, and contralateral block of the lower limbs may prevent early exercise postoperatively.

Our patients were all severely affected by ankylosing spondylitis for decades. As for case 1, the patient had no movement in his spine and had difficulty walking or sitting up from the supine position, and he was always kept on a rigid sitting position with bilateral hip and knee joints fixed at about 90° flexed. With an anticipated difficult airway and impairment of pulmonary function, general anesthesia with tracheal intubation is a big challenge. An ultrasound examination of lower back showed extreme narrowing of his lumbar vertebral spaces. Because calcification of the ligamentum flavum impeded the penetration of ultrasound, epidural space and ‘posterior complex’ could not be identified through lumbar interlaminar space with a paramedian oblique sagital scan described by Karmakar [19]. So a central neuraxial block was not considered.

According to Miller’s Anesthesia, the upper dermatome level of sensory block to T10 is recommended for hip surgery with spinal anesthesia [20]. Recent study demonstrated that a dermatome level up to T12 may satisfy the requirement of surgical anesthesia for hip replacement [21]. The nerves innervating the hip joints derived from the ventral rami of the spinal nerve roots of the lower part of the lumbar plexus (L2-4) and the upper part of the sacral plexus (L4-S1) [22]. The lateral femoral cutaneous nerve from the lumbar plexus (L2-L3), lateral cutaneous branch of iliohypogastric nerve (T12 and L1) and subcostal nerve (T12 thoracic nerve) innervate the area of the superior lateral gluteal region and the proximal lateral thigh that involved in skin incision of posterolateral approach to hip joint. The sacral plexus block technique used by us are based on Taha’s method [11]. The injected local anesthetics will spread between the strong pelvic fascia and the piriformis muscle bathing the entire sacral plexus [23]. More than a sciatic nerve block, this technique can induce an unilateral sacral plexus block by what is equivalent to other paravertebral approaches [24]. Because all other branches of the sacral plexus can be blocked, it is more effective for hip surgery when combined with lumbar plexus block. However, due to the lack of reliable sensory block in dermatome T12 and L1 [25], a combination of lumbar plexus and sacral plexus block failed to provide consistent surgical anesthesia for hip surgery [6, 8, 26, 27]. Several techniques have been introduced to overcome the limitation, such as large dosage of propofol and opioids [8], infiltration at incision [4] and iliac crest point block [9]. However, these techniques were unreliable, and conversion to general anesthesia was required occasionally. It has been reported that a combination of L1 paravertebral block with psoas compartment block and sciatic nerve block could provide reliable surgical anesthesia for partial hip operation [28]. In our report, ultrasound guided T12-L1 PVB was applied to block the subcostal nerve and iliohypogastric nerve, which may also block the superior gluteal cutaneous nerve derived from posterior branch of L1 and L2 spinal nerves through a paravertebral space spread. This complementary block is safe, effective and simple, and will produce sensory blockade covering the surgical incision exceeding to the level above the great trochanter on the superior lateral gluteal region [29].

The potential advantages could be anticipated for our combined peripheral nerves block for THA include avoided stressful awake intubation, eliminated multiple-attempted lumbar puncture, improved hemodynamic stability [27], reduced opioids requirements, decreased PACU discharge time, earlier ambulation and participation in physical therapy programs with less discomfort, and overall improved patient satisfaction [8]. However, there are some setbacks with our novel combination technique for patients with ankylosing spondylitis. Firstly, we need 3 separate needle insertion procedures, and this would take relatively long time consumption which may make the patients discomfort. Secondly, with a relatively large dosage of local anesthetics, the patients may be subject to risk of systemic toxicity or bilateral block from retrograde epidural diffusion which occurred in 9–16% of adult patients [20]. Thirdly, long term immobilization of limbs, and usage of glucocorticoid caused osteoporosis and the concerning bony landmark less clear in the ultrasound screen. The target points were deep and the needle was inserted in a large angulation, this may make it difficult to recognize the tip of the needle. Enough experiences are expected to perform this combination technique. Strategies including injection with small increment and appropriate pressure, and close monitoring should be considered. If contralateral block were detected, we should repeatedly check the blocking level, and frequently monitored the blood pressure, and use fluid infusion or vasopressors to maintain a stable hemodynamic situation.

## Conclusions

The combination of T12 paravertebral block, lumbar plexus block and sacral plexus block may provide reliable surgical analgesia, adequate muscle relaxation, and satisfied postoperative pain control for total hip replacement in patients with ankylosing spondylitis.

## Abbreviations

AS: Ankylosing spondylitis
PBI: Posterior border of the ischium
PVB: Paravertebral block
THA: Total hip arthroplasty
